# Creation of a Novel Biomedical Engineering Research Course for Incarcerated Students

**DOI:** 10.1007/s43683-022-00071-6

**Published:** 2022-06-29

**Authors:** Julie E. Speer, Zoe Elisa Clapacs

**Affiliations:** 1 Department of Biomedical Engineering, Washington University in St. Louis, St. Louis, MO USA; 2 Teaching and Learning Center, A.T. Still University, Mesa, AZ USA

**Keywords:** Prison education, Asynchronous learning, Mentorship, Undergraduate research, Incarcerated students, Engineering education

## Abstract

Options for incarcerated individuals to participate in higher education in prison programs (HEPPs) have expanded in recent years to include courses in science, technology, engineering, and mathematics, however these students remain an underserved population in the United States. Thus, there are opportunities to expand the available offerings, increase the diversity of coursework available by introducing subjects such as biomedical engineering (BME), and include cocurricular and extracurricular activities widely considered critical components of undergraduate training including research experiences. As such, a year-long program was developed to introduce students pursuing a bachelor’s degree in an HEPP through an R1 institution to research principles in BME. This course introduced students to disciplines within BME, offered opportunities to gain research experience as knowledge-creators, and supported engagement with a scientific learning community. Using a student-centered approach, the course was designed to incorporate activities for reflection, goal setting, and dialogue among participants and sought to leverage students’ funds of knowledge and areas of personal scientific interest. This course represents a transferable model for offering BME courses and research-centered opportunities to students enrolled in other HEPPs and an opportunity to promote equity and access in higher education.

**Supplementary Information:**

The online version contains supplementary material available at 10.1007/s43683-022-00071-6.

## Challenge Statement

Despite having only 5% of the world’s population, the United States has 25% of the world’s carceral population and incarcerated individuals are disproportionately people of color and individuals with less educational attainment prior to incarceration.^1–5^ Recent data demonstrate that Black and Latinx individuals are incarcerated at rates nearly 5 and 1.3 times higher than White individuals, respectively, and on average individuals enter prison with less than 11 years of education, more than two years below the Unites States’ national average.^1–4^ There are over 300 higher education in prison programs (HEPPs) in the United States that serve approximately 71,000 students.^6–8^ HEPPs provide opportunities for students to earn GEDs and/or post-secondary degrees and engage in training opportunities.^6,9–13^ However, access to such programs varies substantially by geographical region. For example, Royer et al. reported that while North Carolina has 44 HEPPs, seven states only had one and three states offer none.^6^ HEPPs also differ in a number of ways including the student populations they serve, the type of correctional facility they are affiliated with, instructional format and modes of engagement (e.g., correspondence courses, live broadcast, asynchronous online, or face-to-face), affiliated academic institutions (e.g., public, private, or for profit; two-year or four-year programs) and admission/enrollment processes.^6^ Despite these differences, the majority of HEPPs offer opportunities for students to enroll in courses, engage with training programs and academic services, and earn post-secondary degrees and/or certificates.^6^ Data have demonstrated that HEPPs impact both students and their communities in ways that go beyond reducing recidivism^14–19^ including increasing opportunities for employment/further education upon re-entry,^9,15,17^ promoting positive health outcomes and personal growth,^1,15,20,21^ and children of prison education participants are themselves more likely to attend college.^22^

Due in part to these successful outcomes, the number of HEPPs has generally increased over the past decade and programs that focus on science, technology, engineering, and math (STEM) have followed this trend.^6^ As of the 2018–2019 academic year, at least 63 programs awarded associate or Bachelor of Science degrees and partnerships with both universities and private companies have facilitated a range of STEM training programs (e.g., workshops, seminars, and internships).^6,10,13,23–26^ While the growth of these programs may represent an increase in access to training opportunities, the scope of HEPPs can be limited given that courses are generally offered based on the specialties of instructor volunteers. As such, students enrolled in HEPPs may not be introduced to the same range of disciplines that non-carcerate students are, representing an issue of equity, access, and inclusion. Additionally, while many programs offer extracurriculars,^6^ there is little to no precedent for creating opportunities for incarcerated students to engage in undergraduate research experiences (UREs) as knowledge creators, despite this being considered a critical experience in non-carceral undergraduate training.^27^ Extensive research validates the positive impact participating in mentored research experiences has for students (e.g., development of research and professional skills, retention in STEM, increased GPA, belongingness, and self-confidence); benefits that may be even more significant for students with identities historically underrepresented in STEM and during challenging times such as the COVID-19 pandemic.^28–35^

## Novel Initiative

### Overview of the Two-Semester Course (Course Goals, Student Population, and Instructional Format)

**Figure 1:**
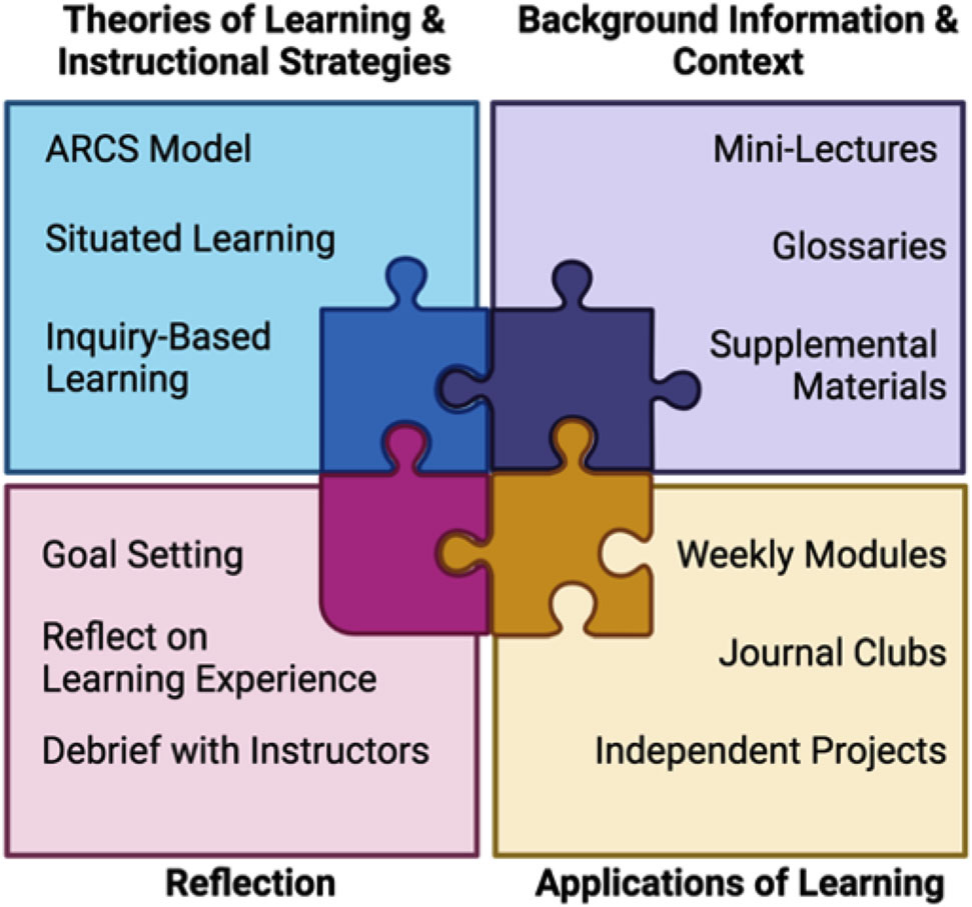
Overview of the model for this BME course-based research experience for incarcerated students. Image created with BioRender.com.

To address these challenges, we developed and implemented a year-long pass/fail course-based research experience (worth 2 credit hours per semester) to introduce students to research principles in biomedical engineering (BME) and to provide opportunities for students to engage in mentored research activities (e.g. planning experiments, analyzing data, appraising scientific literature, contextualizing experimental results based on prior findings, and communicating research outcomes; see Online Resource 1 for syllabi materials). At the time this course was conceptualized, both authors were doctoral students with experience serving as teaching assistants and mentors in research laboratories. Drawing from those experiences and training in evidence-based pedagogy, we designed a two-semester course for students pursuing a Bachelor of Science degree with a concentration in natural sciences and mathematics in an HEPP affiliated with an R1 institution located in the Midwestern United States. Students enrolled in the HEPP were recruited to the course by academic advisors/faculty and five chose to register. Figure 1 identifies specific ways student-centered teaching approaches were used throughout the course to achieve the overarching course goals of increasing scientific literacy and communication skills, supporting students in developing a scientific identity and engaging with a STEM learning community, and promoting an understanding of experimental methods and active research areas in BME.

**Figure 2:**
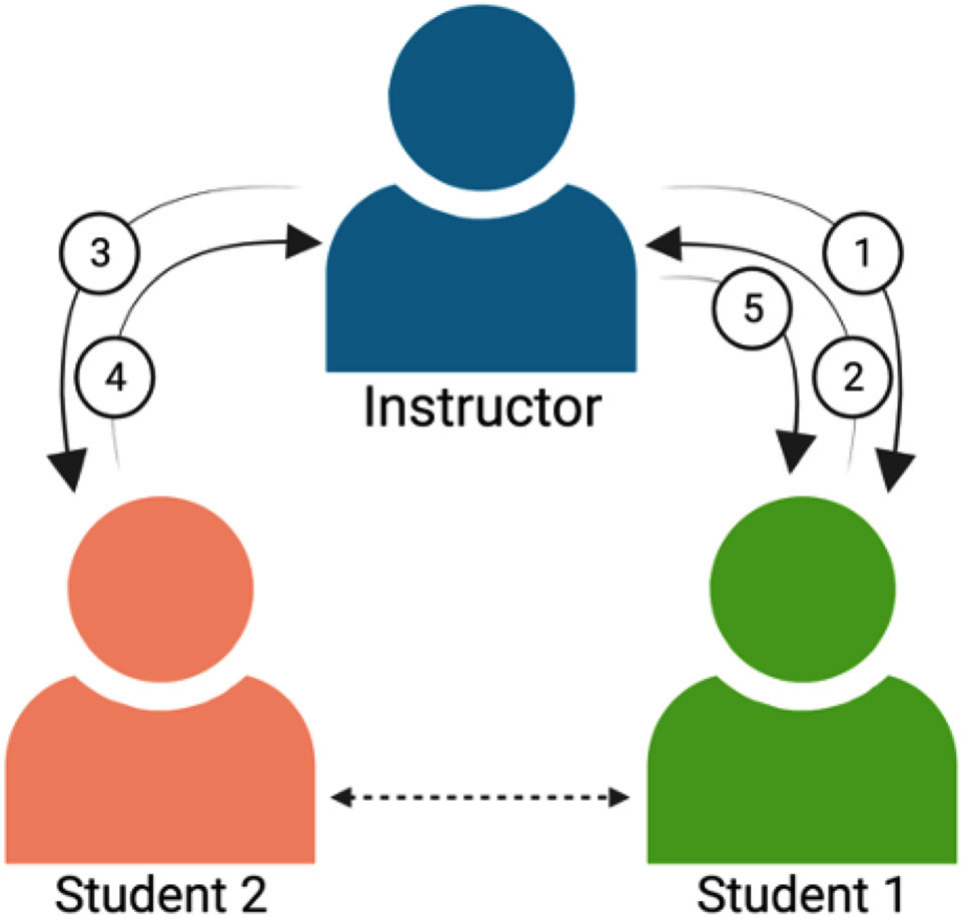
Process for facilitating peer-peer feedback and review. Instructors provided worksheets or documents to students (1) who then submitted their assignments back to the instructors (2). The instructor then sent the document to a peer for review (3) and then the second student submitted the document back to the instructor (4). Finally, the instructor sent the document to the original student. An example of a peer-peer reflection sheet can be found in Online Resource 3 and additional materials are available upon request. Image created with BioRender.com.

Due to the COVID-19 pandemic, the course was offered asynchronously and virtually (remotely) via a learning management system (LMS) which students accessed using tablets issued by the correctional center. Through this platform, students were able to watch short video lectures, receive and turn in assignments, and communicate with instructors. While this technology enabled the course to occur at a time when face-to-face instruction was impossible, several limitations informed our approach to the course design. First, the students’ tablets do not provide direct access to the internet, instead, students must synchronize their tablet at kiosks within the correctional center to upload or download materials. This necessitated direct instructor involvement in facilitating literature searches and providing supplementary materials such as guides on performing calculations and using functions in spreadsheets. Additionally, students are unable to message each other directly using the LMS. Because of this limitation, document transfer between students for peer-peer feedback and collaboration was mediated by instructors (Fig. 2).

### First Semester—Fall 2020

**Figure 3:**
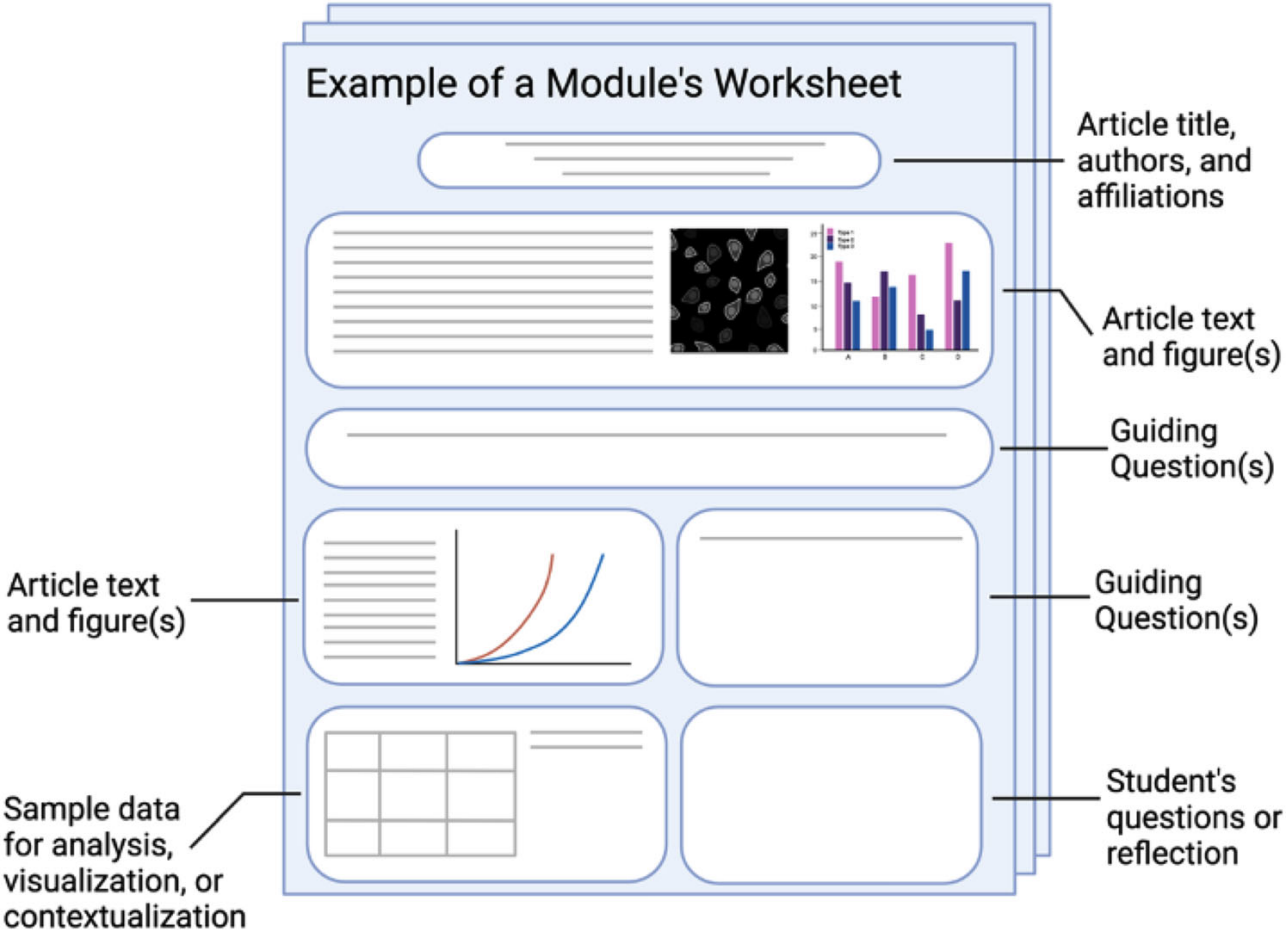
Each module contained activities such as worksheets which provided students opportunities to engage with scientific literature by stepping through the article and answering guided questions related to the methodology, data, and results. The modules also provided students with sample data to analyze, contextualize, and/or visualize and space for reflections. Examples of worksheets from two different modules can be seen in Online Resources 2 and 4 which were used to accompany a discussion about several scientific articles and resources.^53–56^ Additional instructional materials are available upon request. Image created with BioRender.com.

In the first semester of the two-semester program, we focused on developing fundamental research skills and providing individualized feedback to students to emulate the environment of a traditional mentored URE. Instruction was organized into modules (each lasting 1–2 weeks) which consisted of a mini-lecture or slide deck created by the instructors and associated activities such as articles to read, worksheets and reflection prompts to engage with, or data to analyze/visualize. These activities provided opportunities for students to practice newly learned skills, participate in summative assessments, and receive feedback from instructors. During the first few modules, students were introduced to the discipline of BME, the scientific method, the structure of scientific articles, and concepts of data presentation. The remaining modules focused on providing opportunities for students to practice reading and analyzing scientific literature by engaging in asynchronous journal club discussions (Online Resource 1). After watching a mini-lecture which provided an overview of the BME topic(s) addressed in the respective scientific article, students used a glossary and a guided worksheet to read and analyze the article. The worksheet (Fig. 3, Online Resource 2) contained questions to promote critical thinking about the article and to prompt students to contextualize and analyze results. A separate worksheet (Online Resource 3) was used to facilitate peer-peer interactions and provided space for students to reflect on what they learned from the article and aspects they found challenging and particularly interesting. After submitting their own reflections, students would receive one from another student. This promoted a peer-peer dialogue and an opportunity to learn from others’ perspectives. Building upon these experiences, the final weeks of the semester focused on students developing a research question of their own, reading relevant literature, proposing an experiment, and analyzing simulated data. As a final deliverable, students compiled an abstract based on the template for the annual Biomedical Engineering Society (BMES) meeting. Through these activities, students had the opportunity to see themselves as knowledge creators and engage in authentic research activities.

### Second Semester—Spring 2021

**Figure 4:**
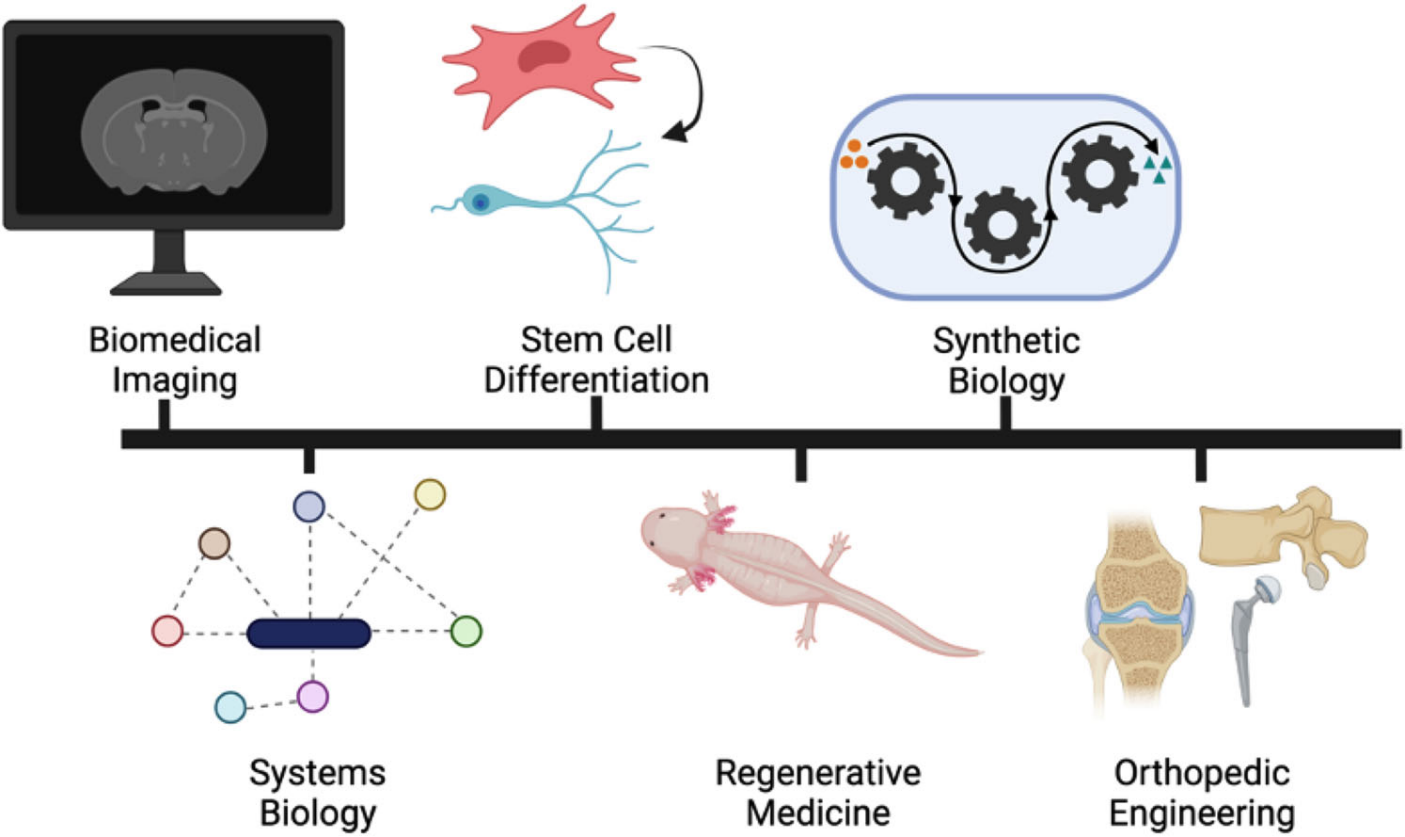
Examples of BME topics covered through modules and journal clubs in the course. Image created with BioRender.com.

In the second semester of the program, students were first invited to reflect on the previous semester and set new personal learning goals. Over the next several weeks, students explored diverse areas of research in the BME disciplines (Fig. 4, Online Resource 1) by engaging in the weekly modules. Like in the first semester, during these modules, students read and interpreted scientific literature, analyzed data, and proposed next steps for the research (Online Resource 4). For the final deliverable in the second semester, each student developed their own module based on an area of interest. The students identified key components of an article and posed reflection questions related to methods, data analysis, and future directions. During each of the final five weeks of the class, a student-generated module was distributed to the class and the responses were returned to the module’s author who then provided feedback to their peers. Finally, each student reflected on the experience of developing the module and sharing it with the class.

## Reflection

While precedent exists for implementing STEM programming and even laboratory courses and hands-on demonstrations within HEPPs,^10,13,24,25,36,37^ to the best of our knowledge, this course represents the first research-focused BME course offered to students enrolled in an HEPP in the United States and a novel opportunity for students to engage in UREs while incarcerated. Given that we were designing a novel program, we undertook significant efforts to identify guiding principles for our instructional approach and to clearly designate course goals and scope. We drew inspiration from constructivism and incorporated evidence-based teaching and learning practices including situated learning, inquiry-based learning, and the ARCS approach (attention, relevance, confidence, satisfaction) into our instructional model in order to build a “brave” and intellectually stimulating learning environment where students could draw from their funds of knowledge and passion for science.^38–43^ Additionally, we modeled a growth mindset approach throughout the course and designed the activities to empower students to express their opinions and to think about their own learning processes. These are approaches we have previously employed in both classroom teaching and mentoring undergraduates face-to-face in the laboratory environment, and we took care to translate them to into this course-based research experience as well.

From our perspectives as instructors, one of the biggest impacts of this course was the opportunity to build a community of researchers within an HEPP that introduced students to the discipline of BME. Given that we were limited by the COVID-19 pandemic to a fully remote and asynchronous format, and had not previously met the students, we knew it would be important to establish a sense of community. To this end, we dedicated time for introductions and goal setting and established lines of multi-directional communication (from instructors to students, students to students, and students to instructors). Students frequently communicated their questions and reflections about the assignments and their interest in receiving more information about a variety of scientific topics. Additionally, students volunteered to support their peers throughout the course by collaborating on assignments and checking on each other during difficult times. We were open with students as well and provided both instruction and mentorship. We conducted frequent check-ins, responded promptly to messages with thorough and thoughtful feedback, requested input from students on the activities and module topics, and shared from our own educational and research experiences. We found that the development of this community fostered creative thinking and a safe environment to challenge oneself. In particular, we observed that students continually pushed themselves to expand their skillsets and to explore scientific topics with great intellectual curiosity. Our experiences corroborate recent work demonstrating that mentorship and engagement in a community may be even more critical during challenging times such as the COVID-19 pandemic. Such work identifies “lessons-learned” and important considerations for providing effective and equitable mentorship and these guidelines will be implemented in this course moving forward.^30,44,45^

During unpredictable times, it can also be even more critical to scaffold the learning experiences and create consistency. We felt that the module design allowed us to provide our students with an experience in which the activities were clearly laid out and through which we were able to model research skills. For example, by working through the guided worksheets, students were able to practice reading and analyzing scientific literature and apply that learning to their independent projects. Additionally, students could draw upon their experiences with the instructor-created modules when developing their own to share with their peers. These guiding principles of the module design are scalable and can be used in future iterations of the course to facilitate discussions on additional BME topics or to teach other research skills.

While we were able to form community during the course and establish a repeatable pattern to the learning experience, the virtual classroom environment nonetheless presented considerable challenges. The asynchronous nature of the course prevented full group discussions and limited the abilities of students to learn with and from one another. Fully remote instruction also limited class activities, practice opportunities, demonstrations, and summative assessments. The technology available to students presented additional challenges. As instructors we had to frequently consider the types of files that the LMS would support and the hardware and software limitations of the students’ tablets. One of the most substantial challenges was that of equipment malfunction. Tablets frequently would not fully synchronize at the kiosks in the housing units and several students’ tablets broke during the course. This meant that students might be unable to send or receive messages, upload or download course materials, and occasionally, files became corrupted during the synching process. When these challenges occurred, we used creative approaches to address the situation and collaborated with other HEPP educators and correctional staff who provided support including delivering hard copies of documents to students and digitizing students’ hand written materials. As such, in the second semester we intentionally designed materials for each module so they could be easily printed and transferred as hard-copies to students if needed. For example, we included PDF versions of the slide decks used for the mini-lectures along with the video recording. We also found that it was helpful to establish policies in the class that provided flexibility to students regarding actions like assignment submission and to work individually with students to accommodate any personal situations which might impact their course experience.

Due to continued interest from both previous and new students, further programming has been developed and is currently being implemented. Students communicated an interest in engaging with additional STEM educational experiences following this course. As such, more informal learning opportunities including a STEM book club were developed and offered virtually over the summer (Summer 2021). During the 2021–2022 academic year, face-to-face instruction has become possible again, which has substantially reduced difficulties of the asynchronous environment and presented an opportunity to teach hands-on laboratory skills. It is important, however, that as we conceptualize future BME courses and educational offerings that such programs be developed in partnership with incarcerated students, HEPPs, and departments of corrections, and should take care to create supportive and intellectually challenging environments that do not underestimate students’ capabilities.^13,46^ An important part of that process is seeking out and implementing feedback from course participants. While in the first year of the course we frequently requested feedback from students, work to systematically investigate the student experiences using a mixed-methods approach is currently ongoing. That study will seek to determine the efficacy of the course(s), quantify student learning outcomes, and catalogue student perspectives. Data from such an educational research study will be used to inform future directions of this program. In the future study, Likert scale questions will be used to collect quantitative data from both HEPP students who have participated in the BME course-based research experiences and non-carcerate students who are participating in UREs. These data will be compared in order to determine the degree to which this course supports incarcerated students in developing technical and transferable research skills learned through a traditional URE. Additionally, both closed- and open-ended questions will be used to collect qualitative responses from HEPP students related to their experiences in the class, their short and long-term goals, and the outcomes of the program.

Expanding the course-based research opportunities has required additional human resources. We have recruited several new BME graduate student facilitators in order to offer the course described herein again and to develop an advanced course for students who had previously completed this introductory program. Onboarding additional instructors for current and future iterations of this course has also provided an opportunity for us to reflect on our own experiences and the impact teaching in this program has had on us as educators. While we both previously held roles as research mentors and teaching assistants, becoming fully immersed in the design and implementation of this course as instructors of record has been an incredible learning opportunity. Our experiences serving as instructors while doctoral students corroborate findings from prior literature—rather than detracting from our research goals, designing and teaching this course contributed to our professional development as researchers, educators, and mentors.^47–49^ We learned about ourselves and our own teaching philosophies and gained translational skills such as practicing techniques for project and classroom management and creative problem solving. Throughout the year we also learned much from, and were inspired by, our students. Being part of this course provided the opportunity for us to learn from our students’ experiences, their learning goals, scientific interests, and zeal for continual learning. Importantly, conceptualizing and participating in this course has empowered us to continue to serve as change agents towards addressing the structural racism present in both the justice and educational systems, implementing high impact, equitable, and inclusive learning environments, and creating cultures of justice, equity, diversity, and inclusion (JEDI) within the scientific and educational communities. Nadkarni et al. have similarly reported that prison education initiatives can have a profound positive influence on the students as well as the educators themselves and can contribute to the development of empathy and interest in social justice issues, particularly for earlier career scientists.^49^

## Conclusion

Over the 2020–2021 academic year we designed and implemented a course for incarcerated students that scaffolded learning with activities authentic to traditional UREs including discussing scientific literature, developing research plans, and interpreting and analyzing data. This was intended to serve as an opportunity for students to develop technical and transferable skills, participate in a scholarly community, and to contribute to developing a “prison-to-STEM pipeline”^9–13^ that will benefit individuals and the scientific community at large. The pedagogical model and the materials presented herein (Online Resources 1–4) can be easily adapted to provide instruction on additional or alternative topics and to meet students where they are based on their own learning goals, previous experiences, and scientific training. As such, this course represents a transferable model for teaching BME courses and research-centered opportunities to incarcerated students enrolled at other institutions (see the National Directory from the Alliance for Higher Education in Prison for a full list of programs in the United States^50^) and an opportunity to promote equity and inclusion in higher education.

## Citation Diversity Statement

Data demonstrate that citation bias exists—minority scholars are often under-cited in relation to the number of published articles in a particular discipline.^51,52^ We recognize the harmful impacts of this bias and we have made efforts to reference literature that reflects diversity of thought as well as gender, race, ethnicity, and other factors.

## Supplementary Information

Below is the link to the electronic supplementary material.

Supplementary file1 (PDF 192 kb)

Supplementary file2 (PDF 84 kb)

Supplementary file3 (PDF 56 kb)

Supplementary file4 (PDF 80 kb)

## Data Availability

Course materials available upon request.

## References

[1] Nowotny, K. M., R. K. Masters, and J. D. Boardman. The relationship between education and health among incarcerated men and women in the United States. *BMC Public Health*. 10.1186/s12889-016-3555-2 27586136 10.1186/s12889-016-3555-2 PMC5009667

[2] Pettit, B., and C. Gutierrez. Mass incarceration and racial inequality. *Am J Econ Sociol*. 10.1111/ajes.12241 10.1111/ajes.12241 PMC9540942 36213171

[3] Nellis, A. The color of justice: racial and ethnic disparity in state prisons. In: The Sentencing Project. 2021. http://www.sentencingproject.org/publications/color-of-justice-racial-and-ethnic-disparity-in-state-prisons/.

[4] Ewert, S., B. L. Sykes, and B. Pettit. The degree of disadvantage: incarceration and inequality in education. *Ann Am Acad Pol Soc Sci*. 10.1177/0002716213503100

[5] Walmsley, R. World prison population list. In: World Prison Brief. 2015. http://www.prisonstudies.org/sites/default/files/resources/downloads/world_prison_population_list_11th_edition_0.pdf.

[6] Royer, C. E., E. L. Castro, E. Cortes-López, et al. The landscape of higher education in prison 2018–2019. 2020. http://www.higheredinprison.org. Accessed 12 Oct 2021.

[7] Wilson, M., R. Alamuddin, and D. Cooper. Unbarring access: a landscape review of postsecondary education in prison and its pedagogical supports. 2019. http://www.sr.ithaka.org/publications/landscape-review-postsecondary-education-in-prison/. Accessed 13 Oct 2021.

[8] Gorgol, L. E., and B. A. Sponsler. Unlocking potential: results of a national survey of postsecondary education in state prisons. Institute for Higher Education Policy. 2011. http://www.files.eric.ed.gov/fulltext/ED521128.pdf. Accessed 12 Oct 2021.

[9] Halkovic, A. Redefining possible: re-visioning the prison-to-college pipeline. *Equity Excell Educ*. 10.1080/10665684.2014.959284

[10] Horns, J. J., N. Nadkarni, and A. Anholt. How repeated exposure to informal science education affects content knowledge of and perspectives on science among incarcerated adults. *PLoS ONE*. 10.1371/journal.pone.0233083 32442217 10.1371/journal.pone.0233083 PMC7244156

[11] Gewin, V. Moving from prison to a PhD. *Nature*. 10.1038/d41586-019-03370-1 33122834 10.1038/d41586-019-03370-1

[12] EDC and partners awarded federal grant to promote pathways to STEM careers for people who are or were incarcerated. 2019. http://www.edc.org/edc-partners-awarded-federal-grant-promote-pathways-stem-careers-incarcerated. Accessed 26 Oct 2021.

[13] Nadkarni, N. M., and J. S. Morris. Baseline attitudes and impacts of informal science education lectures on content knowledge and value of science among incarcerated populations. *Sci Commun*. 10.1177/1075547018806909

[14] Castro, E. L. Racism, the language of reduced recidivism, and higher education in prison: toward an anti-racist praxis. *Crit Educ*. 10.14288/ce.v9i17.186357

[15] Delaney, R., R. Subramanian, and F. Patrick. Making the grade: developing quality postsecondary education programs in prison. 2016. http://www.vera.org/downloads/publications/making-the-grade-postsecondary-education-programs-in-prison.pdf. Accessed 14 Oct 2021.

[16] Simpkins, B. College inside: a case study of the design and implementation of a successful prison college program. *New Dir Community Coll*. 10.1002/cc.20140

[17] Bozick, R., J. Steele, L. Davis, *et al*. Does providing inmates with education improve postrelease outcomes? A meta-analysis of correctional education programs in the United States. *J Exp Criminol*. 10.1007/s11292-018-9334-6

[18] Esperian, J. H. The effect of prison education programs on recidivism. *J Correct Educ* 61(4):316–334

[19] Davis LM, Bozick R, Steele JL, et al. Evaluating the effectiveness of correctional education: a meta-analysis of programs that provide education to incarcerated adults. RAND Corporation; 2017.

[20] Delaney R, Smith L, Soroui J. Understanding educational aspiration among people in prison. 2019. http://www.voced.edu.au/content/ngv%3A83964. Accessed 14 Oct 2021.

[21] Evans, D. The elevating connection of higher education in prison: an incarcerated student’s perspective. *Crit Educ*. 10.14288/CE.V9I11.186318

[22] Ross, J., and R. Gangi. Education from the inside out: the multiple benefits of college programs in prison. 2009. http://citeseerx.ist.psu.edu/viewdoc/summary?doi=10.1.1.175.2624. Accessed 14 Oct 2021.

[23] Byrne, C. P. Maths in prison. *J Prison Educ Reentry*. 10.15845/jper.v2i2.720

[24] Bretl, T. Teaching undergraduate courses on robotics and control in prison. *Mech Eng*. 10.1115/1.2018-SEP8

[25] Hardin, J., K. Haushalter, and D. Yong. Turning STEM education inside-out: teaching and learning inside prisons. *Sci Educ Civ Engagem* 12(2):82–88

[26] Next chapter: helping formerly incarcerated individuals find work and succeed in tech. http://www.slack.com/blog/news/next-chapter-a-pilot-program-aiming-to-help-formerly-incarcerated-individuals-find-work-and-succeed-in-tech. Accessed 14 Oct 2021.

[27] Schowen, K. B. Research as a critical component of the undergraduate educational experience. In: Assessing the value of research in the chemical sciences: report of a workshop. Washington, DC; 1998. pp. 73–81.

[28] Zydney, A. L., J. S. Bennet, A. Shahid, *et al*. Impact of undergraduate research experience in engineering. *J Eng Educ*. 10.1002/j.2168-9830.2002.tb00687.x

[29] McIntee, F., K. R. Evans, J. M. Andreoli, et al. Developing undergraduate scientists by scaffolding the entry into mentored research. *Scholarsh Pract Undergrad Res*. 10.18833/spur/2/1/5 10.18833/spur/2/1/5 PMC6261301 30506020

[30] Speer, J. E., M. Lyon, and J. Johnson. Gains and losses in virtual mentorship: a descriptive case study of undergraduate mentees and graduate mentors in STEM research during the COVID-19 pandemic. *CBE Life Sci Educ*. 10.1187/cbe.20-06-0128 33734867 10.1187/cbe.20-06-0128 PMC8734387

[31] Balster, N., C. Pfund, R. Rediske, *et al*. Entering research: a course that creates community and structure for beginning undergraduate researchers in the STEM disciplines. *CBE Life Sci Educ*. 10.1187/cbe.09-10-0073 20516356 10.1187/cbe.09-10-0073 PMC2879377

[32] Seymour, E., A.-B. Hunter, S. L. Laursen, *et al*. Establishing the benefits of research experiences for undergraduates in the sciences: first findings from a three-year study. *Sci Educ*. 10.1002/SCE.10131

[33] Fechheimer, M., K. Webber, and P. B. Kleiber. How well do undergraduate research programs promote engagement and success of students? *CBE Life Sci Educ*. 10.1187/cbe.10-10-0130 21633064 10.1187/cbe.10-10-0130 PMC3105922

[34] Bruthers, C. B., and M. L. Matyas. Undergraduates from underrepresented groups gain research skills and career aspirations through summer research fellowship. *Adv Physiol Educ*. 10.1152/advan.00014.2020 32880486 10.1152/advan.00014.2020

[35] Pagano, T., A. Ross, and S. B. Smith. Undergraduate research involving deaf and hard-of-hearing students in interdisciplinary science projects. *Educ Sci*. 10.3390/educsci5020146

[36] Mickelwait, K. Giving back: the prison university project. In: UC Berkeley: MCB Transcript. 2017. http://www.mcb.berkeley.edu/news-and-events/transcript/giving-back-prison-university-project. Accessed 22 Oct 2021.

[37] Wang, L. Chemistry behind bars. *Chem Eng News* 85(43):17–22

[38] Richards, R., C. Powell, J. Hammack, et al. Mentoring undergraduate research. *Undergrad Res Creat Endeav*. 2014; Paper 1.

[39] Keller, J. M. Development and use of the ARCS model of instructional design. *J Instr Dev* 10(3):2–10

[40] Fernandez, O. E. How constructivism can boost success in STEM fields for women and students of color. In: Constructivist Education in an Age of Accountability edited by D. W. Kritt. Cham, 2018, pp. 113–126.

[41] Hunter, A.-B., S. L. Laursen, and E. Seymour. Becoming a scientist: the role of undergraduate research in students’ cognitive, personal, and professional development. *Sci Educ*. 10.1002/sce.20173

[42] Besar, P. H. S. N., and P. H. Binti. Situated learning theory: the key to effective classroom teaching? *HONAI Int J Educ Soc Polit Cult Stud* 1(1):49–60

[43] McCright, A. M. Enhancing students’ scientific and quantitative literacies through an inquiry-based learning project on climate change. *J Scholarsh Teach Learn* 12(4):86–102

[44] Stephenson-Hunter, C., S. Franco, A. Martinez, *et al*. Virtual summer undergraduate mentorship program for students underrepresented in medicine yields significant increases in self-efficacy measurements during COVID-19 pandemic: a mixed methods evaluation. *Heal Equity*. 10.1089/heq.2021.0060 10.1089/heq.2021.0060 PMC8665814 34909539

[45] Cameron, K. A., L. A. Daniels, E. Traw, *et al*. Mentoring in crisis does not need to put mentorship in crisis: realigning expectations. *J Clin Transl Sci*. 10.1017/cts.2020.50834192060

[46] Castro, E. L., M. Brawn, D. E. Graves, *et al*. Higher education in an era of mass incarceration: possibility under constraint. *J Crit Scholarsh High Educ Stud Aff* 1(1):13–31

[47] Shortlidge, E. E., and S. L. Eddy. The trade-off between graduate student research and teaching: a myth? *PLoS ONE*. 10.1371/journal.pone.0199576 29940027 10.1371/journal.pone.0199576 PMC6016899

[48] Brandt, P. D., S. Sturzenegger Varvayanis, T. Baas, *et al*. A cross-institutional analysis of the effects of broadening trainee professional development on research productivity. *PLoS Biol*. 10.1371/journal.pbio.3000956 34264929 10.1371/journal.pbio.3000956 PMC8282014

[49] Nadkarni, N. M., J. Horns, J. M. Chen, *et al*. Reversing the Lens on Public Engagement with Science: Positive Benefits for Participating Scientists. *Bioscience*. 10.1093/biosci/biac003

[50] National Directory. Alliance for Higher Education in Prison. https://www.higheredinprison.org/national-directory.

[51] Dworkin, J. D., K. A. Linn, E. G. Teich, *et al*. The extent and drivers of gender imbalance in neuroscience reference lists. *Nat Neurosci*. 10.1038/s41593-020-0658-y 32561883 10.1038/s41593-020-0658-y

[52] Bertolero, M. A., J. D. Dworkin, S. U. David, *et al*. Racial and ethnic imbalance in neuroscience reference lists and intersections with gender. *BioRxiv*. 10.1101/2020.10.12.336230

[53] Bridgen, D. T., B. V. Fearing, L. Jing, *et al.* Regulation of human nucleus pulposus cells by peptide-coupled substrates.*Acta Biomater*. 2017;55:100–108. 10.1016/j.actbio.2017.04.019 28433788 10.1016/j.actbio.2017.04.019 PMC5536110

[54] Wu, M., Y. Chen, H. Xia, *et al.* Transcriptional and proteomic insights into the host response in fatal COVID-19 cases. *Proc Natl Acad Sci*. 2020;117(45):28336–28343. 10.1073/pnas.2018030117 33082228 10.1073/pnas.2018030117 PMC7668053

[55] Gilchrist, C. L., E. M. Darling, J. Chen, *et al.* Extracellular matrix ligand and stiffness modulate immature nucleus pulposus cell-cell interactions. *PloS one*. 2011;6(11):e27170. 10.1371/journal.pone.0027170 22087260 10.1371/journal.pone.0027170 PMC3210142

[56] The UniProt Consortium. UniProt: the universal protein knowledgebase in 2021. *Nucleic Acids Res*. 2021;49(D1):D480–D489. 10.1093/nar/gkaa1100 10.1093/nar/gkaa1100 PMC7778908 33237286

