# Supplementary material for: Creation of a Novel Biomedical Engineering Research Course for Incarcerated Students
Source: Biomed Eng Educ. 2022 Jun 29;2(2):157–65. doi: 10.1007/s43683-022-00071-6 (PMC9244394; doi:10.1007/s43683-022-00071-6)
Supplement: Supplementary file 1 — Supplementary file1 (PDF 192 kb) [file 43683_2022_71_MOESM1_ESM.pdf]

Truncated Course Syllabi and Schedules[1]

Examples of Course Policies and Expectations Included in the Full Syllabi (Adapted in part from the University and HEPP Policies)

Enrollment and Grading

- No pre-requisites are required for this course
- This course is 2-credits and will be graded as pass/fail
- Late assignments will be accepted and will be considered as complete.
- Multiple submissions can be made for an assignment, all submissions will be reviewed.
- A passing grade will be achieved by completing the majority of course assignments
- Collaboration is critical in science and thus you may collaborate with the mentors, other students, and/or tutors. However as in science, it is important that you acknowledge your collaborators' contributions to your work or the sources you have used.
- All materials necessary for an assignment will be uploaded to the LMS. To prepare for the weekly module, please watch the video and/or read the lecture slides or supplementary materials. These will provide background and instructions for the weekly assignments.

Course Goals and Expectations for Participation and Engagement

- This independent study course will provide opportunities to engage in research, analyze and summarize laboratory data, and discuss the research communicated by other scientists.
- This course prioritizes trying your best over getting the right answer the first time, learning from and sharing with your peers, reflecting on your personal learning process, and a peer-review process.
- Students are expected to respect that each student is coming to the course with different prior experiences (both life and scientific) and may have taken different coursework. Therefore, when engaging with others students, instructors, or tutors, constructive feedback is appropriate but hurtful language is not acceptable.
- The goal of this course is to engage in scientific activities and we will place an emphasis on the research process and your experience of engaging in research. Therefore, assignments will be marked as complete or incomplete rather than graded on a numeric scale.
- Best practices to maximize learning include but are not limited to: taking notes, reviewing instructor feedback on graded work, and asking questions.
- Participation in the course includes reading the weekly materials, working on the assignments, and taking part of the peer-review activities.
    - If you are unable to participate due to challenging circumstances (examples include but are not limited to, COVID-19, other health concerns, or other circumstances that pose a challenge to your ability to learn and work), please let the instructor(s) know and they will work with you to individually.
    - If you have any concerns about the course or interactions with other students during course activities please speak with an instructor, your academic advisor, or HEPP administrators.
- In order to affirm each person’s gender identity and lived experiences, it is important that we ask and check in with others about pronouns. This simple effort can make a profound difference in a person’s experience of safety, respect, and support.

---

[1] Syllabi were truncated to remove policies and other information that may identify the program or participants, as well as to promote clarity, brevity, and ease of future adaptation.

- There is also an expectation for civil discourse in the virtual classroom. Civility is demonstrated through mutual respect, for people, their knowledge, and their ideas. Civility requires cooperation, tolerance, acceptance, inclusiveness, kindness, courtesy, and patience. It is expressed not only in the words we choose, but in our tone, demeanor, and actions. While disagreement will, and should, occur in a collegiate setting, open communication, intellectual integrity, mutual respect for differing viewpoints, freedom from unnecessary disruption, and a climate of civility are values we embrace and expect. No kind or degree of incivility will be tolerated in the virtual classroom.

Learning Goals and Objectives

- *Goal 1: Provide an introduction to the foundations of scientific research and the practice of science*
    - By the end of the course, students will be able to…
        - Understand and explain the importance keeping scientific notes and records
        - Understand and explain why it is important to be meticulous with methodology
        - Understand and explain the difference between raw data and processed data
        - Evaluate the validity of a scientific claim and explain the process of doing so
        - Explain the significance of a research question
        - Generate a hypothesis based on evidence, and design an experiment to test that hypothesis
        - Identify research areas within the field of biomedical research and be able to describe examples of research questions that pertain to each
        - Design a research experiment informed by scientific literature and provide and incorporate peer-feedback.

- *Goal 2: Provide an introduction to data and interpretation*
    - By the end of the course, students will be able to…
        - Understand and apply the scientific definition of significance
        - Understand and explain the meaning of a p-value
        - Apply a t-test and understand when it is and is not appropriate to use
        - Design an experiment
        - Understand and explain how the method of data collection can influence the proper interpretation of data
        - Use statistics to analyze research data and evaluate research claims

- *Goal 3: Provide an introduction to the foundations of scientific communication*
    - By the end of the course, students will be able to…
        - Format and present data to clearly convey its interpretation
        - Know and differentiate between the typical parts of a primary research paper
        - Understand and explain the difference between different types of scientific articles
        - Recognize the format of, and be able to create, a conference abstract or poster
        - Create data visualizations that communicate research findings

Course Schedules

Semester 1 (Fall 2020)

Module	Topic	Associated Activities and Assignments
1	Personal introductions and goal setting; introduction to the scientific method	Complete introduction document, set specific goals for the course
2	Organization and communication in science; introduction to laboratory notebooks and scientific communication formats including posters and abstracts	Research methods activity, read an example of an abstract and a poster, reflection activity
3	Data literacy and presentation, introduction to statistical significance (what does it mean, how do we test for it?)	Worksheet on data literacy and data presentation using example data
4	Deconstructing Scientific Manuscripts	Response prompts to scientific literature sections
5	Journal Club Discussion # 1	Guided reading with worksheet, peer discussion worksheet
6	Experimental design, data analysis part 2	Statistical analysis of laboratory data
7	Journal Club Discussion # 2	Guided reading with worksheet, peer discussion worksheet
8	Developing a hypothesis	Hypothesis worksheet, independent research proposal week 1 (picking a topic area and starting to generate a hypothesis)
9	Independent research proposal week 2	Compare and contrast results and methods from literature
10	Independent research proposal week 3	Proposing an experiment, generating sample data
11	Independent research proposal week 4	Summarizing results, conclusions, address limitations in study/future directions
12	Wrap up	Compile abstract, feedback on class, future learning goals

Semester 2 (Spring 2021)

Module	Topic	Associated Activities and Assignments
1	Introduction to Spring Semester (including reflection on Fall Semester, goal setting for Spring Semester. overview of Spring Semester)	Complete reflection worksheet
2	Biomedical Imaging	Complete Week 2 worksheet
3	Systems Biology	Complete week 3 worksheet, brainstorm for independent Project
4	Biomaterials Engineering	Complete week 4 worksheet, pick article for independent project
5	Stem Cell Differentiation	Complete week 5 worksheet, peer feedback/reflection
6	Synthetic Biology	Complete week 6 worksheet, draft literature section of independent project

7	Orthopedics	Complete week 7 worksheet, draft data interpretation questions for independent project
8	Accessibility and Adaptive Engineering	Complete week 8 worksheet, peer feedback and reflection
9	Regenerative Medicine	Complete week 9 worksheet, finalize independent project
10	Student Module 1	Complete worksheet from student project 1
11	Student Project 2	Complete worksheet from student project 2
12	Student Project 3	Complete worksheet from student project 3
13	Student Project 4	Complete worksheet from student project 4
14	Student Project 5	Complete worksheet from student project 5
15	Wrap Up	Wrap up, resubmit assignments having incorporated peer feedback
