# Supplementary material for: Creation of a Novel Biomedical Engineering Research Course for Incarcerated Students
Source: Biomed Eng Educ. 2022 Jun 29;2(2):157–65. doi: 10.1007/s43683-022-00071-6 (PMC9244394; doi:10.1007/s43683-022-00071-6)
Supplement: Supplementary file 2 — Supplementary file2 (PDF 84 kb) [file 43683_2022_71_MOESM2_ESM.pdf]

Example 1 of a Guided Reading Worksheet

This worksheet was used for the second journal club of the first semester (Fall 2020) and accompanied Bridgen, Devin T., et al. "Regulation of human nucleus pulposus cells by peptide-coupled substrates." Acta biomaterialia 55 (2017): 100-108.

Guided reading worksheet: Fill out this worksheet as you read through the article. This will be turned in to the instructors but will not be shared with peers.

Title:

1. What is the title setting you up to expect from this paper?

Authors:

1. First look at the authors list. Who is the first author?
2. Who is the senior author on the paper (the last author)?
3. Do all the authors come from the same institution?

Abstract:

1. From the abstract of the paper, what is the overarching hypothesis of this paper?
2. What do the authors say are the major findings of the paper?

Other pre-article details:

1. How long after the paper was submitted, was it accepted? After that how long until it was published?
2. What funding sources supported this work?

Introduction:

1. What integrin receptors have been previously shown in NP cells to facilitate NP cell-ECM interactions?
2. What extracellular matrix proteins have been found in the NP ECM?
3. Please note that reference 21 is the paper we just read (Gilchrist, Christopher L., et al. "Extracellular matrix ligand and stiffness modulate immature nucleus pulposus cell-cell interactions." *PloS one* 6.11 (2011): e27170.).
4. Why do the authors say they are interested in using peptides rather than full proteins?
5. What is the overall objective of this study?
6. What was does this new research expand on previous findings?

Materials and Methods:

1. What is the source of this study's NP cells? Is this the same or different from the Gilchrist+ 2011 paper?
2. Which peptides do the authors use to functionalize the polyacrylamide gels? (Note: the letters in the sequence show the amino acids in that sequence)
3. What method do the authors use for measuring the stiffness of their gels? How many samples do they take?
4. How many cells do these authors define as a cell cluster?
5. On what days did the authors measure sGAG production?
6. What metrics do the authors quantify to define the NP cell phenotype?

Results:

1. What do the authors show with Figure 1A.
2. What trends do you observe in Figure 1B and 1C?
3. Based on Figure 2 - What two stiffnesses of polyacrylamide do the authors use in this study? Does conjugated peptide vary by substrate stiffness?
4. In Figure 3, which conditions promote NP cell behaviors that are similar to or greater than that seen on soft laminin?
5. In Figure 3, for their statistical analysis, which laminin condition are the authors comparing the other data to?
6. Based on Figure 4, what would you recommend to another scientist as the best option for promoting NP cell behaviors?

Discussion:

1. How did the authors select the peptides they would use in their study?
2. What cell behaviors do the authors indicate are demonstrative of non-degenerative NP cells?
3. Gilchrist+ 2011 showed that NP cells can sense and respond to both ligand and substrate stiffness. In what ways does this paper further that finding?
4. What steps for future work do the authors say they or other people could take?
5. What were the overall conclusions the authors discuss in the paper?
