# Supplementary material for: Creation of a Novel Biomedical Engineering Research Course for Incarcerated Students
Source: Biomed Eng Educ. 2022 Jun 29;2(2):157–65. doi: 10.1007/s43683-022-00071-6 (PMC9244394; doi:10.1007/s43683-022-00071-6)
Supplement: Supplementary file 3 — Supplementary file3 (PDF 56 kb) [file 43683_2022_71_MOESM3_ESM.pdf]

Example of a Peer-Peer Feedback Worksheet

Below is an example of the worksheet used by students to reflect on their experience reading a scientific article and to share their perspectives and opinions.

Reflection on the Paper*
* This will be shared with one of your peers. Turn in the top part, and then when you get a response from your peers (emailed by an instructor), then answer the bottom part.

1. What was your experience of reading this paper?

2. What was something you learned from reading this paper?

3. What was a finding, figure, or subplot of a figure you liked? Why did you like this? What did you find interesting about it?

4. What was something you found confusing? How could they have made this clearer to a reader?

5. Do you agree with authors' major conclusions? Why or why not?

6. What is a follow-up question you have or if you were working with these authors, what experiment would you want to run next given these results?

7. What was something you are taking away from reading this paper? How can you apply something you learned to your own academics or science?

After you received a document to review answer the below questions:

Having read the responses to the above questions from another student, please answer the following:

1. In what ways was your peer's experience of reading the paper similar or different from yours?

2. What was something you learned from reading your peer's responses?

3. Did reading the responses from a peer change your perspective on the paper or the findings?

4. What does their follow-up question make you think of? How might they test that question?
