# Supplementary material for: Creation of a Novel Biomedical Engineering Research Course for Incarcerated Students
Source: Biomed Eng Educ. 2022 Jun 29;2(2):157–65. doi: 10.1007/s43683-022-00071-6 (PMC9244394; doi:10.1007/s43683-022-00071-6)
Supplement: Supplementary file 4 — Supplementary file4 (PDF 80 kb) [file 43683_2022_71_MOESM4_ESM.pdf]

Example 2 of a Guided Reading Worksheet

The questions below accompanied a module on systems biology during the second semester (Spring 2020) and were included in a document that guided students through reading Wu, Meng, et al. "Transcriptional and proteomic insights into the host response in fatal COVID-19 cases." Proceedings of the National Academy of Sciences 117.45 (2020): 28336-28343 and results from the UniProt Database: "UniProt: the universal protein knowledgebase in 2021." Nucleic acids research 49, no. D1 (2021): D480-D489.

Please read before starting: This module will present findings related to COVID-19 and describes patients who had fatal cases. COVID-19 has impacted everyone and their families in different ways and we want to be respectful of that. If for any reason you are uncomfortable with reading this article about COVID-19, you do not have to. The instructors can provide an alternative article. Please just reach out to them directly if you would like to read the alternative paper.

If you wish to proceed reading this article, please go to the next page.

1. Why might it be important for the authors to do quality control before proceeding?

2. Why might it be important for the raw data to be made publicly available?

3. What are some observations you made regarding the patient's cases plotted in Figure 1?

4. The volcano plot in Figure 2A gives us a visual of the upregulated genes (in orange), downregulated genes (in cyan) and not significant (ns) genes in grey. Each dot = 1 gene. Approximately what percentage of the total genes would you roughly estimate falls into each of these categories? Did you expect this many genes to be differentially regulated by COVID-19 infection when you started reading the paper?

5. The structure of the diagram is like the one in the lecture slides. Each row is a gene and each column is a patient. Based on these heat maps what do you notice about the pattern of upregulated genes between the COVID+ patients and control patients? Did every COVID+ patient have the same value for every gene?

6. What pathways were enriched in both the lung and colon tissues of patients with COVID-19? Please list 2 gene sets or pathways which had at least 60 differentially regulated genes in that set for lung tissue and colon tissue?

7. If you were on this team of researchers, what is a question you might ask next? Could you answer the question with the current data, or do you need to collect new data?

8. Suppose your teammate comes to you with the following gene expression data. They tell you that the table shows the absolute expression of each gene in the respective groups.

Patient #	Gene 1 (PTX3)		Gene 2 (DEFB125)	
	Non-COVID Lung	COVID Lung	Non-COVID Lung	COVID Lung
1	10	40	200	3
2	7	38	159	4
3	11	56	157	5
4	5	47	240	3
5	8	39	180	6
6	9	42	192	7
7	10	43	174	5
8	9	41	186	5
9	12	37	191	3

On average, what is the relative gene expression (FC = COVID Lung/Non-COVID Lung) for the two genes?

a) PTX3 = 5, DEFB125 = 0.02
b) PTX3 = 0.2, DEFB125 = 45
c) PTX3 = 0.05, DEFB125 = 45
d) PTX3 = 0.05, DEFB125 = 10

9. Your teammate wants to know what statistical test they should run to see if to determine if there is a difference in PTX3 expression between the COVID+ and COVID- patients in the table above. What test should they run?

a) ANOVA
b) Linear Regression
c) No Stats are Needed
d) T-test

10. You run the test for them and determine that the p-value is 1.23736E-11. Based on these findings your teammate says that there are differences in PTX3 expression between the COVID+ and COVID- groups? Based on these findings, can your teammate say that there are differences in COVID+ patient's expression of PTX3 and DEFB125?

11. Now that you've done this analysis for your teammate, you are curious what this protein is! So you look it up in the Uniprot Database (online encyclopedia of genes). It tells you the following: "PTX3 = Pentraxin-related protein PTX3; Plays a role in the regulation of innate resistance to pathogens, inflammatory reactions, possibly clearance of self-components and female fertility."

And it tells you this gene has the following GO terms, are any of these standing out to you as interesting in the context of COVID?:

"(1->3)-beta-D-glucan binding, complement component C1q complex binding, identical protein binding, virion binding, extracellular matrix organization, inflammatory response, innate immune response, negative regulation by host of viral exo-alpha-sialidase activity, negative regulation by host of viral glycoprotein metabolic process, negative regulation by host of viral process, negative regulation of exo-alpha-sialidase activity, negative regulation of glycoprotein metabolic process, negative regulation of viral entry into host cell, neutrophil degranulation, opsonization, ovarian cumulus expansion, positive regulation of nitric oxide biosynthetic process, positive regulation of phagocytosis, response to yeast."

12. How could you use synthetic biology to answer questions you have related to COVID or another topic?

13. Do you have questions about this paper or the lecture that you would like to ask?
